# The evolution of basal mantle structure in response to supercontinent aggregation and dispersal

**DOI:** 10.1038/s41598-021-02359-z

**Published:** 2021-11-25

**Authors:** Xianzhi Cao, Nicolas Flament, Ömer F. Bodur, R. Dietmar Müller

**Affiliations:** 1 Frontiers Science Center for Deep Ocean Multispheres and Earth System; Key Lab of Submarine Geosciences and Prospecting Techniques, MOE, and College of Marine Geosciences, Ocean University of China, Qingdao, 266100 China; 2 EarthByte Group, School of Geosciences, The University of Sydney, Sydney, NSW Australia; 3 GeoQuEST Research Centre, School of Earth and Environmental Sciences, University of Wollongong, Northfields Avenue, Wollongong, NSW 2522 Australia

**Keywords:** Geodynamics, Geology, Geophysics, Tectonics

## Abstract

Seismic studies have revealed two Large Low-Shear Velocity Provinces (LLSVPs) in the lowermost mantle. Whether these structures remain stable over time or evolve through supercontinent cycles is debated. Here we analyze a recently published mantle flow model constrained by a synthetic plate motion model extending back to one billion years ago, to investigate how the mantle evolves in response to changing plate configurations. Our model predicts that sinking slabs segment the basal thermochemical structure below an assembling supercontinent, and that this structure eventually becomes unified due to slab push from circum-supercontinental subduction. In contrast, the basal thermochemical structure below the superocean is generally coherent due to the persistence of a superocean in our imposed plate reconstruction. The two antipodal basal thermochemical structures exchange material several times when part of one of the structures is carved out and merged with the other one, similarly to “exotic” tectonic terranes. Plumes mostly rise from thick basal thermochemical structures and in some instances migrate from the edges towards the interior of basal thermochemical structures due to slab push. Our results suggest that the topography of basal structures and distribution of plumes change over time due to the changing subduction network over supercontinent cycles.

## Introduction

Two nearly antipodal LLSVPs at the base of the mantle separated by a ring of seismically fast anomalies characterize the basal mantle^1^. The origin of the LLSVPs is unclear and widely debated due to limited knowledge of the lower mantle^2,3^. LLSVPs are proposed to be closely associated with Earth’s surface evolution. For example, they are thought to be the reservoir for ocean island basalts^4^, responsible for the degree-two geoid anomaly^5^, and to affect the velocity of tectonic plates^6^. The relationship between LLSVPs and plate motion is controversial, with some models implying that LLSVPs have remained stable over long time periods, while others suggest that they could be shaped by subducted slabs that have sunk into the deep mantle^6–11^. Characterizing the plate-basal mantle relationship is important to better understand Earth’s long-term evolution.

Seismologists have progressively refined the imaging of the LLSVPs with growing data sets and improving modelling techniques^12^. The Pacific LLSVP is generally mapped as a horizontally rounded structure that is slightly elongated in the East–West direction, while the African LLSVP is narrower and stretches in the North–South direction from the northern Atlantic Ocean to the southwest Indian Ocean^12,13^. Together, the LLSVPs cover ~ 20–30% area of the CMB^14,15^. Forward travel time and waveform modeling reveal that the African Anomaly is about ~ 1,000 km wide and ~ 1,200–1,300 km high beneath southern Africa^13,16^, the Pacific structure is ~ 740 km high in the west and ~ 340–650 km high in the east^17^. The lateral extent of the Pacific LLSVP is not well constrained due to the scarce coverage of seismic stations around that structure. Hernlund and Houser^18^ found a nearly bi-modal distribution of shear velocity (*V*_*s*_) in the basal mantle, which is absent in the compression wave velocity (*V*_*p*_) distribution. They proposed that the lower *V*_*s*_ peak is likely associated with chemically distinct material, which extends to 700 km above the core-mantle boundary (CMB), and represents ~ 2% of the volume of the mantle. By taking the − 1% *V*_*s*_ contour as the LLSVP boundary, Burke et al.^14^ mapped the height of the Pacific and African LLSVPs above the CMB as 1,384 km and 1,814-km high, respectively, together representing 1.6% of the volume of the mantle. The margins of the LLSVPs exhibit a wide range of topographies, varying from shallow dipping to near vertical^12,19^. The large variation of the detailed LLSVP morphology inferred by different mantle tomographic models reflects both the scarcity of available data and the unclear definition of LLSVPs. Steep margins were identified at some portions of the western^12^ and eastern^20^ margins of the African structure, and also at the southern^21^, eastern^19^, and western^22^ margins of the Pacific structure, which were suggested to be compositional boundaries^20^. The anticorrelation between shear wave and bulk sound velocity anomalies^23^ also suggests that the LLSVPs are chemically distinct from the surrounding mantle. LLSVPs could possibly contain primordial material accumulated in Earth’s early history by ancient differentiation processes^24^, or alternatively contain eclogitic materials from subducted oceanic crust^25–27^. The LLSVPs are therefore sometimes called thermochemical piles.

Earth's surface evolution is characterized by tectonic motions of rigid plates. The majority of continental lithosphere can aggregate into one body to form a supercontinent. The generally accepted supercontinents include Nuna (ca. 1600–1400 Ma), Rodinia (ca. 900–700 Ma), Pangea (ca. 320–200 Ma), and possibly another short-lived supercontinent called Pannotia (ca. 620–600 Ma)^28–30^. It has been proposed that successive supercontinents can form by introversion (i.e., young interior oceans open during supercontinent breakup and close again to assemble the subsequent supercontinent, while the old superocean external to the previous supercontinent persists) or extroversion (the relatively old ocean external to the previous supercontinent closes to assemble the subsequent supercontinent)^31^. The transition between subsequent supercontinents generally involves both end-member models^32^.

Recent 3D mantle flow models with self-consistent plate generation show that Earth’s surface and mantle (especially the shallow part) is one dynamic system^33^. However, whether the LLSVPs, located at the base of the mantle, respond to changes in surface plate configuration remains controversial. Reconstructed eruption sites of Large Igneous Provinces (LIPs)^14,34^ and kimberlites^35^ over the past ~ 320 Myr, and major hotspots^14^ lie above the edge of the LLSVPs, which has led to the concept of the “plume generation zone”^14^, and implies that LLSVPs could be stationary, non-deforming and insensitive to plate motions over the past ~ 320 Myr and possibly for the whole Phanerozoic^14,35^. In contrast, mantle convection calculations driven by kinematic surface boundary conditions obtained from plate motion models suggest that sinking slabs result in deformation and motion of basal thermochemical structures^2^, and predict present-day basal structures in first-order agreement with seismic observations^8,11^. Statistically steady-state 3D mantle flow modelling suggests that the basal mantle could be characterized by one basal structure in the superocean hemisphere (degree-1 convection planform) during the assembly stage of a supercontinent, and two antipodal basal structures (degree-2 convection planform) shortly after the aggregation of a supercontinent, with one structure forming under the supercontinent in response to circum-supercontinent subduction and a larger antipodal structure under the superocean^36^. Forward mantle models, combined with the spatial match between post-Pangea circum-Pacific subduction and present-day cold anomalies at the CMB, and the evidence for fast P- and S-wave anomalies resembling slabs extending to the lower mantle^37^, suggest a potential link between the configuration of plates at Earth’s surface and the thermochemical structure of the deep mantle^38,39^.

**Figure 1:**
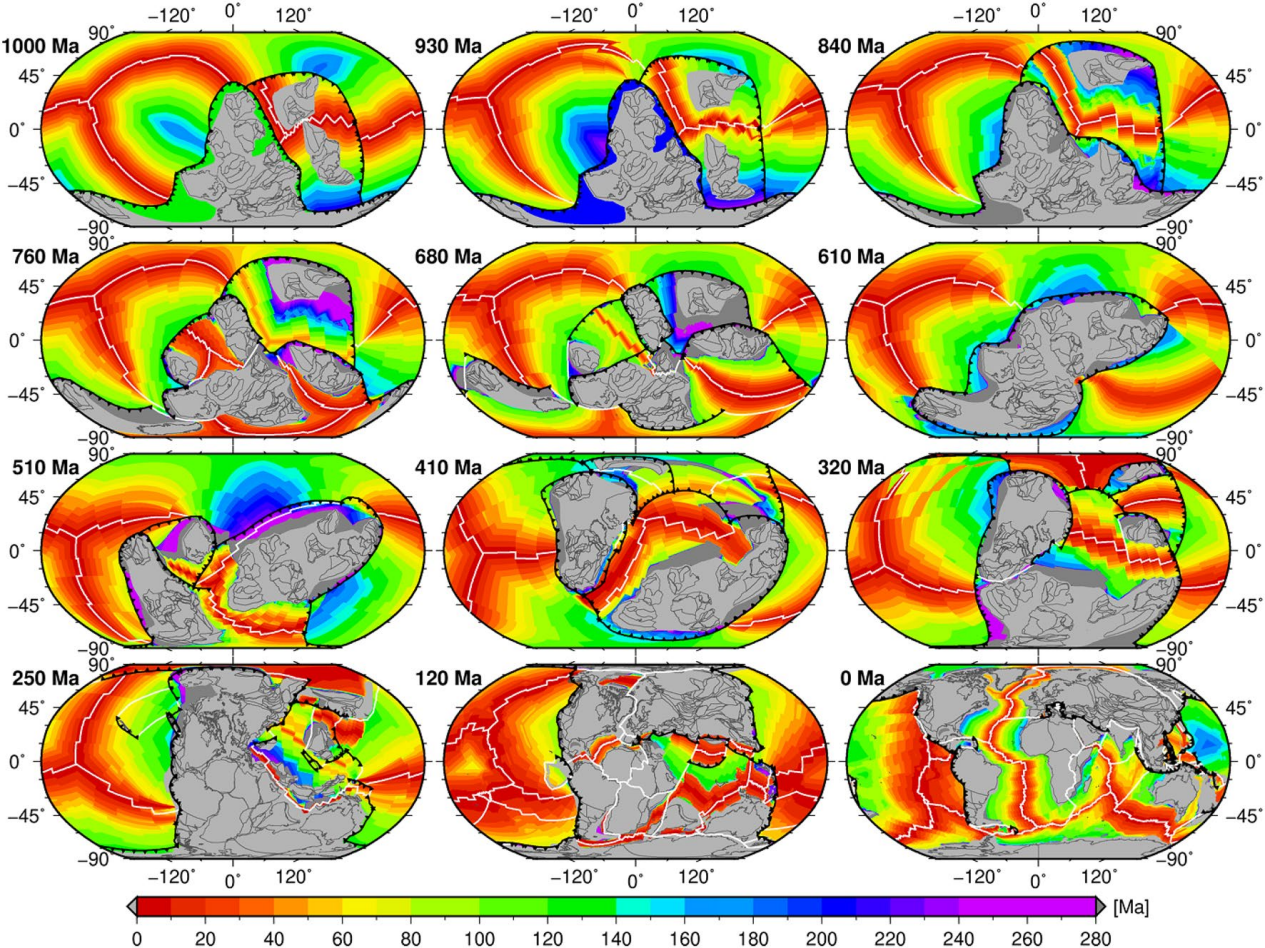
Synthetic plate reconstructions since 1000 Ma (plate motion model NNR from Cao et al.^38^). The 1000–250 Ma part is synthetic, and the 250–0 Ma part is from Young et al.^40^. Three supercontinents assemble by introversion, and exist between 900 and 800 Ma (first supercontinent), 620–600 Ma (second supercontinent) and 320–200 Ma (Pangea), respectively. The colour scale shows ocean floor age. Mid-ocean ridges are shown as white solid lines, subduction zones as toothed black lines. This figure was created using the Generic Mapping Tools version 4.5.5 (https://www.generic-mapping-tools.org/)^85^.

Here we investigate the plate-basal mantle relationship through analysing a recent global mantle flow model (case NNR_WU from ref.^38^), in which an end-member plate tectonic model is applied as an evolving kinematic boundary condition over one billion years (Fig. 1). In the initial condition of the mantle flow model at 1200 Ma, the basal thermal boundary layer includes a 113-km-thick layer of material (2% of the volume of the mantle) that is 1% denser than ambient mantle, which could reflect that LLSVPs are composed either of primordial materials^24^ or of previously subducted oceanic crust^25^. This initial condition is the simplest geometrically, although mantle convection would have formed basal thermochemical structures before one billion years ago. In the model, the tectonic configuration at 1000 Ma is applied as a warm-up phase between 1200 and 1000 Ma, so that the basal layer is no longer laterally homogeneous by 1000 Ma. The 1-Gyr plate tectonic model consists of a synthetic plate motion history between 1,000–250 Ma, which is smoothly connected to an existing palaeogeographical plate reconstruction between 250 and 0 Ma^40^ (detailed in “Methods” section). Three supercontinents are considered between 900 and 800 Ma (to mimic Rodinia^28^), 620–600 Ma (to mimic Pannotia^30^), and 320–200 Ma (Pangea), respectively. The supercontinents predominantly breakup and reassemble by introversion. A circum-(super)continent subduction girdle exists at all times in the plate reconstructions, largely separating Earth into a superocean hemisphere and a (super)continent hemisphere. Since the reconstructions are synthetic before 250 Ma, here we focus on the deformation and migration of basal thermochemical structures and plumes as a response to the breakup and assembly of supercontinents, as opposed to the predicted evolution of the structure of the mantle.

## Results

**Figure 2:**
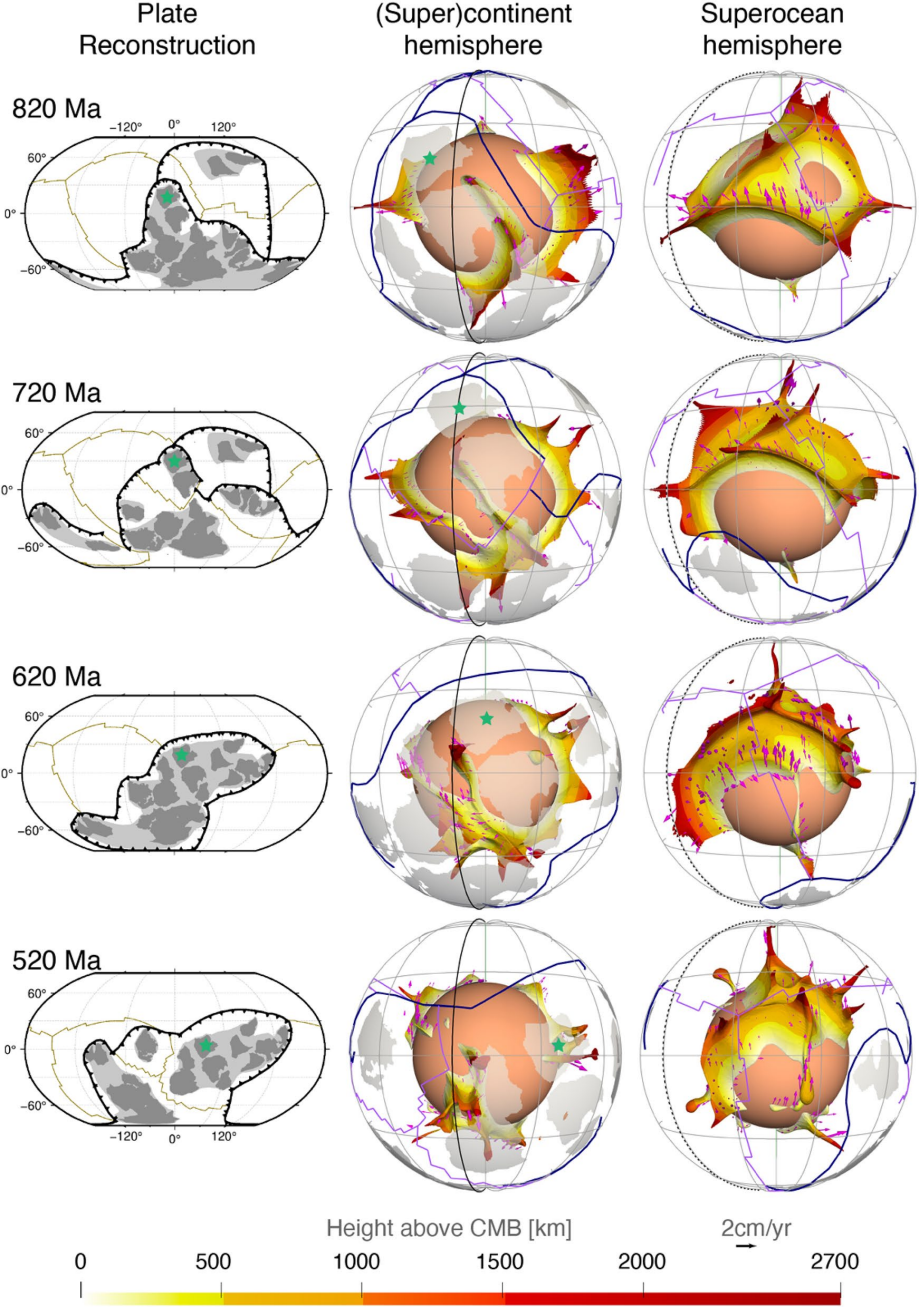

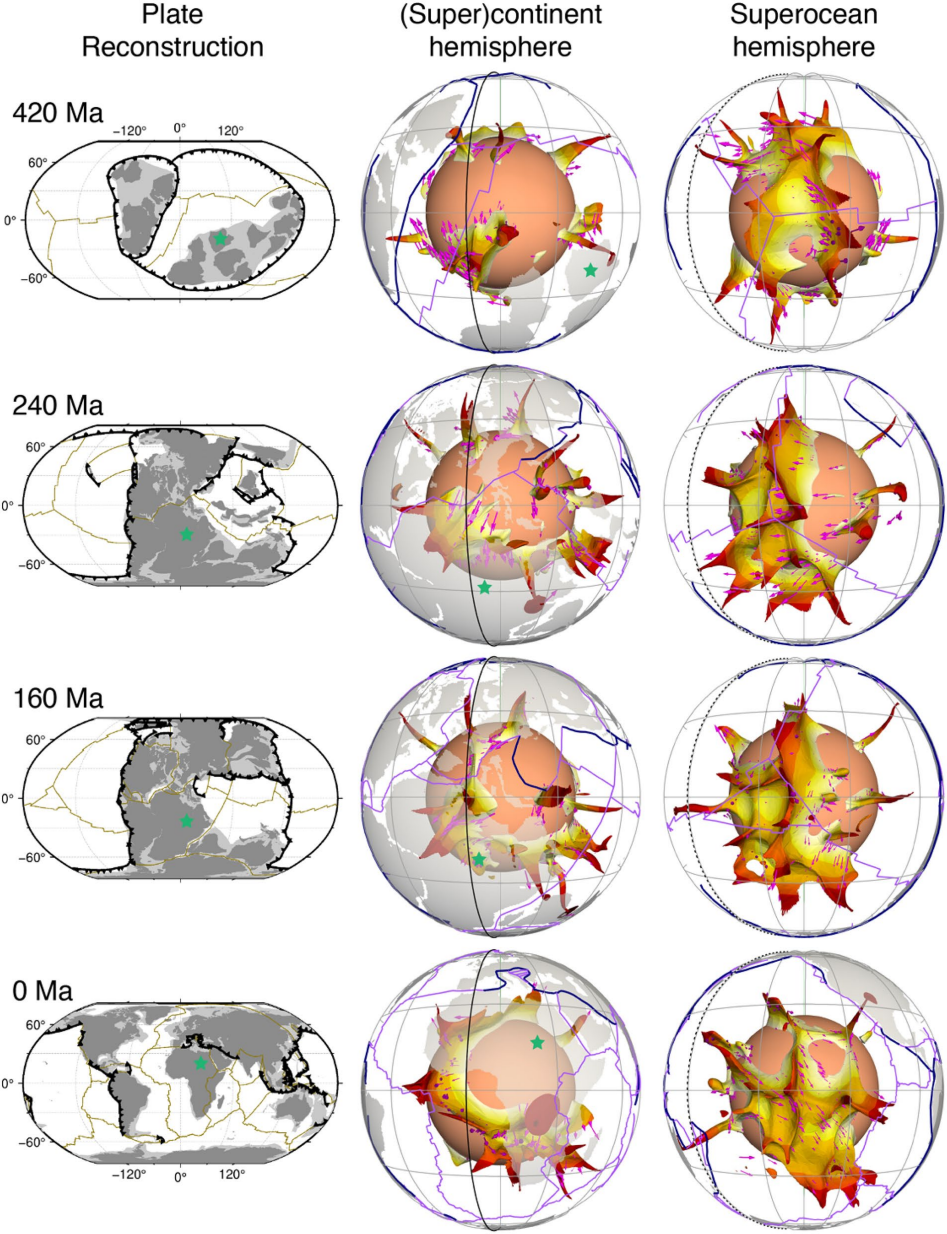
Sequence of snapshots of our plate reconstructions and predicted height of basal thermochemical structures (defined as mantle 310 K hotter than layer average) above the CMB in each hemisphere. In the left panel, dark grey polygons are the major cratons reconstructed before 250 Ma, and light grey polygons denote continental crust. In the middle and right panels, dark blue lines denote subduction zones, purple lines denote ridges and transforms, and magenta arrows show the velocity of the thermochemical structures. The grey great circles are graticules. The solid and dashed black meridians denote longitudes 0º and 180º, respectively. The green star marks a location for reference between 2 and 3D reconstructions. This figure was created using the Generic Mapping Tools version 4.5.5 (https://www.generic-mapping-tools.org/)^85^ and ParaView version 5.8.1 (https://www.paraview.org/)^86^.

**Figure 3:**
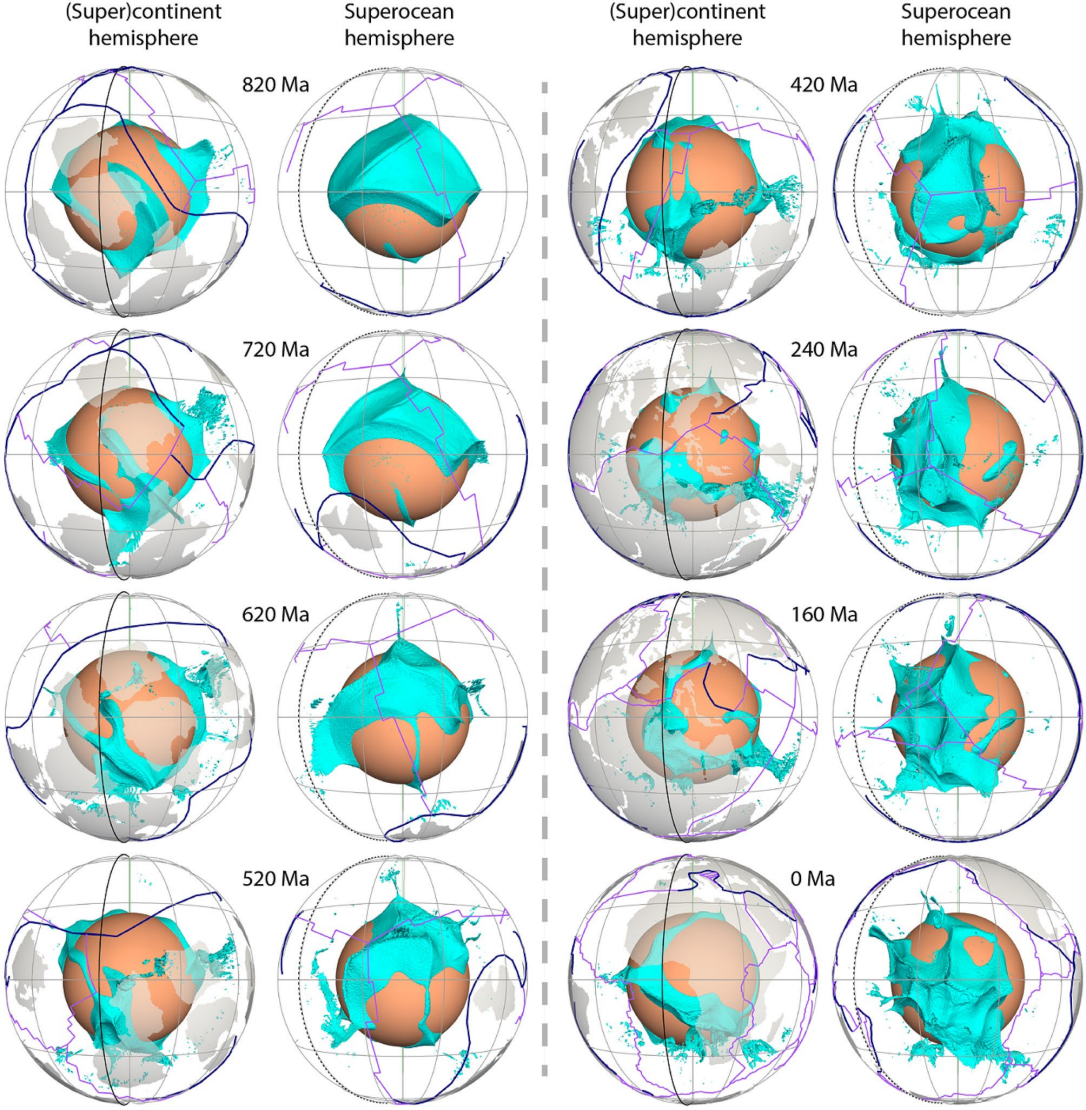
Time-dependent shape of the chemically-distinct basal material (cyan isosurface enclosing mantle consisting of at least 50% dense material) above the CMB. The solid and dashed black meridians are longitudes 0º and 180º, respectively. The view directions for the two hemispheres are the same as in Fig. 2. This figure was created using ParaView version 5.8.1 (https://www.paraview.org/)^86^.

**Figure 4:**
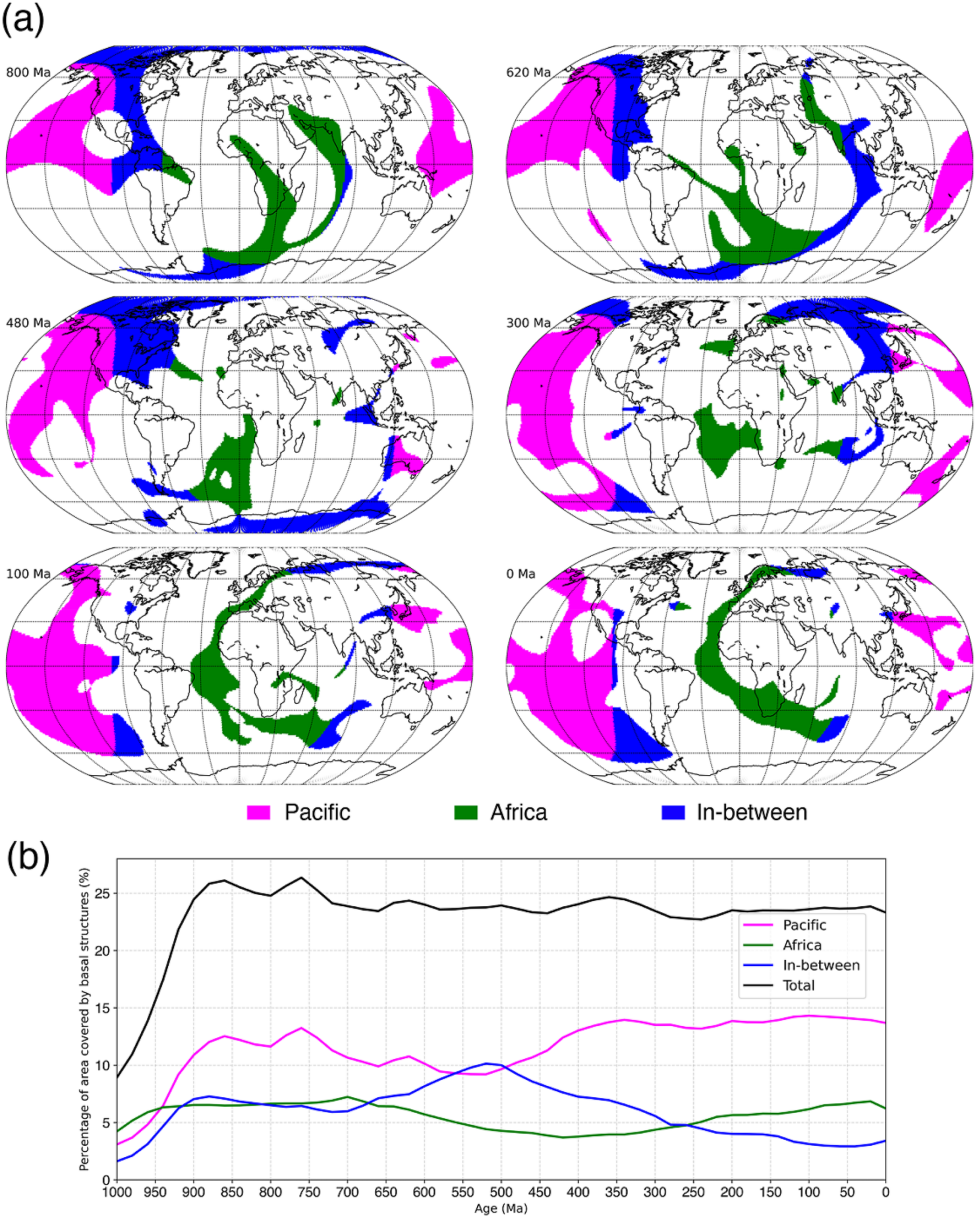
Basal thermochemical structures (mantle 310 K hotter than layer average) at 2677 km depth. **(a)** The thermochemical structures are separated into three equal-area parts: below present-day Africa (centred on lon/lat = 11° E/0°, with a radius of 70.5°), below the Pacific Ocean (centred on lon/lat = 169° W/0°, with a radius of 70.5°), and the ring in-between. **(b)** Percentage of each of the three equal-area parts covered by thermochemical structures in **(a)**. This figure was created using matplotlib version 3.2.2 (https://matplotlib.org/)^87^.

In the flow model, basal thermochemical structures are hotter than ambient mantle. Mantle 310 K hotter than layer average (Fig. 2) generally coincides with mantle consisting of at least 50% dense material (Fig. 3). Relatively little dense material is entrained above ~ 2000 km depth (Supplementary Fig. S1), so that mantle plumes are best represented using temperature anomalies (Fig. 2). Here we use high temperature anomalies to represent both basal thermochemical structures and mantle plumes. Descending slabs along the circum-superocean subduction girdle penetrate the lower mantle and fall on the uniform dense layer during the early model period, leading the basal dense material to flow towards mantle upwelling areas (Supplementary Animations 1 and 2). The heat flux history at the CMB indicates that the model reaches an equilibrium by ~ 840 Ma (Supplementary Fig. S2), so in the following we focus on the last 840 Myr. The predicted present-day surface heat flux (~ 40 TW without radioactive heat production on the continental crust, which is 6–8 TW, Jaupart et al.^41^) and CMB heat flux (~ 13 TW) are consistent with constraints: 47 ± 2 TW at the surface (Davies and Davies^42^) and 5–13 TW at the CMB (Jaupart et al.^41^). The predicted basal thermochemical structures cover ~ 23–26% area of the CMB (Fig. 4), and are generally 600–800 km high (Fig. 2), which is in first order agreement with seismic tomographic studies^14,15,17,18^.

The thermochemical structures in our model deform and migrate over time as they adjust to changes in surface plate configuration (Figs. 2 and 4, Supplementary Animations 1 and 2). Slabs sinking from the subduction girdle largely separate predicted basal thermochemical structures into two hemispheres. The superocean hemisphere is generally characterized by a coherent, horizontally rounded and large basal thermochemical structure over time, while the (super)continent hemisphere is characterized either by a smaller and narrower and horizontally elongated structure or by a few small, segmented structures (Figs. 2 and 4). This is because the supercontinent covers a smaller area than the superocean. Furthermore, there are often more slabs in the interior of the (super)continent hemisphere due to subduction between plates that tends to squeeze basal thermochemical structures into a long and narrow shape, especially during supercontinent assembly. For example, the model predicted present-day North–South elongated shape of the African LLSVP is a consequence of slabs from the convergence between Africa and Eurasia pushing the denser layer westward (Supplementary Fig. S3 and Supplementary Animation 1). The model mantle structure confirms the predominance of two main structures at present-day.

We find that the supercontinent basal thermochemical structure alternates between segmented and unified configurations during a supercontinent cycle, with these configurations postdating the supercontinent dispersal and assembly by a few tens of million years. For example, after Pangea assembles at ~ 320–300 Ma, subducting slabs along the subduction girdle continuously sink to the lower mantle and sweep smaller thermochemical structures into a continuous structure in the supercontinent hemisphere by ~ 120–100 Ma (Figs. 2 and 4). Similarly, the structure in the (super)continent hemisphere is split into smaller structures by ~ 500–480 Ma (Figs. 2 and 4), due to slabs sinking from ~ 700 Ma—these slabs are associated with the breakup of the first supercontinent by the formation of small ocean basins in the (super)continent hemisphere (Figs. 1 and 2). The time lag between surface plate configuration and basal mantle configuration is ~ 200 Myr, which is consistent with the expected slab sinking time from Earth’s surface to the deep mantle in similar flow models^11^ and by jointly considering global tomographic models and tectonic reconstructions^43,44^. The second supercontinent only forms for 20 Myr (620–600 Ma), which is too short for a reorganization of thermochemical structure to occur.

**Figure 5:**
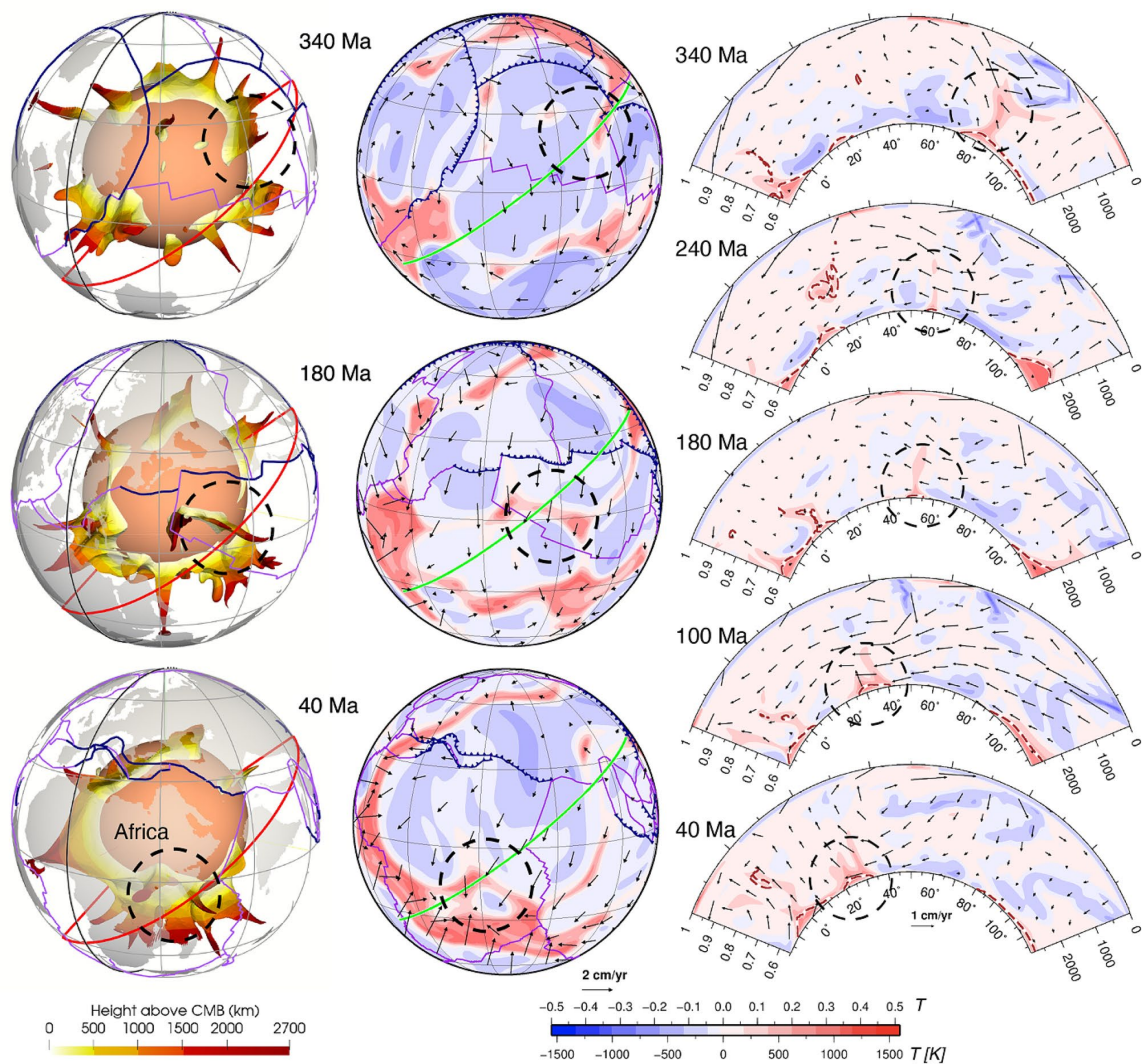
Example of material exchange between mantle structures from different hemispheres. The left panel shows the basal thermochemical structures (310 K hotter than layer average), the middle panel shows temperature anomaly at 2677 km depth, and the right panel shows temperature anomalies (temperature field with layer average removed) along a Northeast-trending cross-section (red and green lines respectively in the left and middle panels). A small thermochemical structure (marked by black dashed circles) is carved out from the superocean structure and subsequently merges with the (super)continent structure. The solid black meridian in the left panel denotes longitude 0º. In the right panel, the brown dashed contours indicate 50% concentration of dense material. The numbers on the left of the cross-section denote non-dimensional radius, and the numbers on the right of the cross-section denote depth (km). The numbers above the colour scale denote non-dimensional temperature, and the numbers below the colour scale denote dimensional temperature. This figure was created using the Generic Mapping Tools version 4.5.5 (https://www.generic-mapping-tools.org/)^85^ and ParaView version 5.8.1 (https://www.paraview.org/)^86^.

The superocean basal thermochemical structure migrates to high latitudes from ~ 650 Ma before it moves back to an equatorial position at ~ 300 Ma (Fig. 4a). As a consequence, between ~ 650 Ma and ~ 350 Ma basal thermochemical structures cover a smaller proportion of the superocean realm, and a larger proportion of the area between the superocean (“Pacific”) and supercontinent (“African”) realms (Fig. 4b). We observe that the thermochemical structures in the superocean and supercontinent realms exchange material several times. For example, a few small structures are separated from the superocean structure between 340 and 260 Ma (such as the structure marked by dashed circles in Fig. 5) due to an episode of trench retreat above it. The smaller structures are then pushed southwestward and eventually merge with the eastern boundary of the (super)continent structure (see around Africa at 40 Ma in Fig. 5). This is similar to tectonic terranes rifted from a major plate and merged with another plate (e.g., Tethys terranes rifted from Gondwana that eventually joined Laurentia^45^). This also indicates the splitting or merging of a thermochemical structure may occur in response to push by sinking slabs.

**Figure 6:**
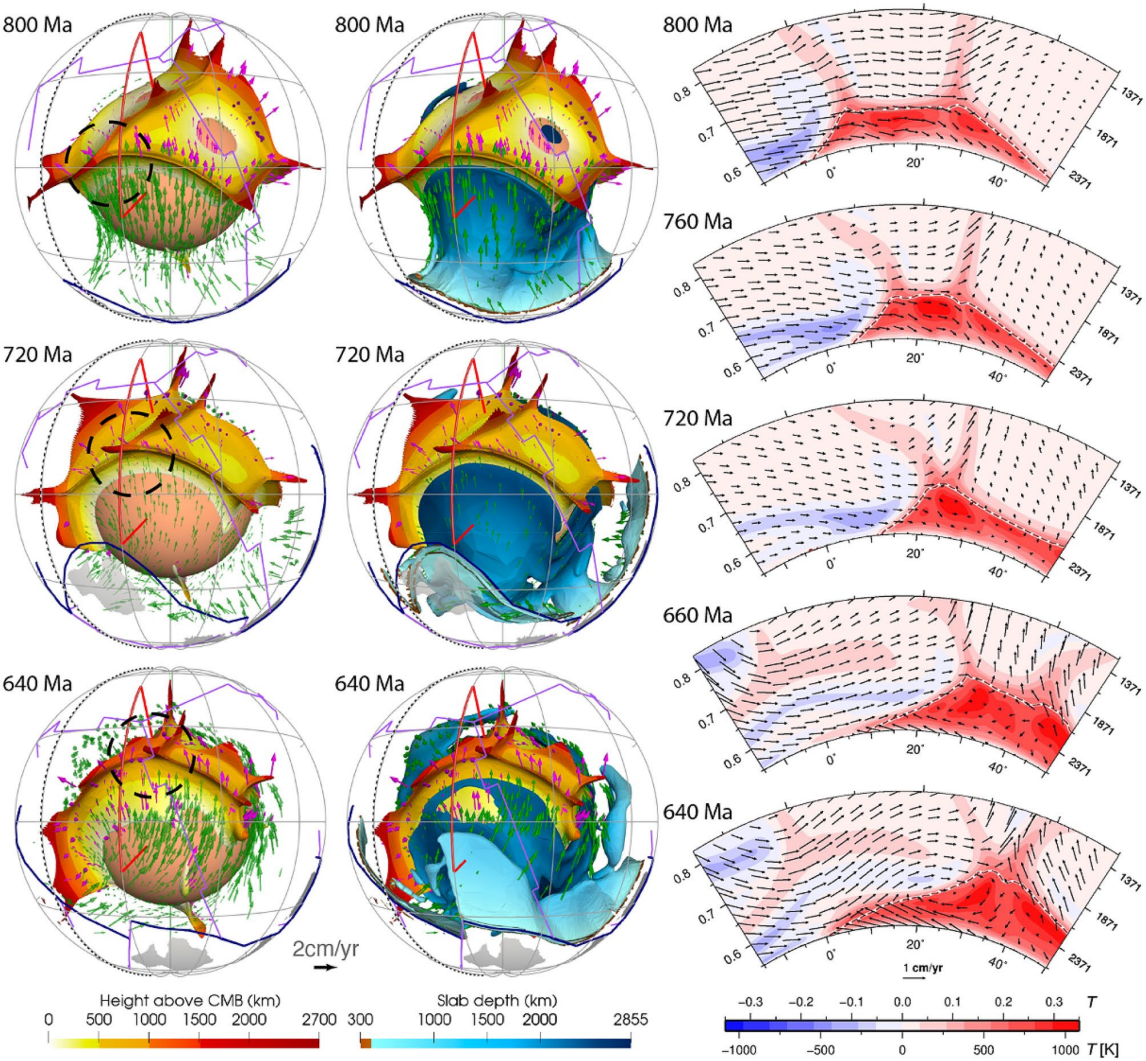
Plume migration on top of the superocean thermochemical structure between 800 and 640 Ma. The left panel shows the thermochemical structures. The middle panel shows both the thermochemical structures and slabs. The dashed black meridian in the left and middle panels denotes longitude 180º. The right panel shows temperature anomaly (temperature field with layer average removed) along a North–South-trending cross-section (red lines in the left and middle panels). Symbols are as in Fig. 2. This figure was created using the Generic Mapping Tools version 4.5.5 (https://www.generic-mapping-tools.org/)^85^ and ParaView version 5.8.1 (https://www.paraview.org/)^86^.

Our model predicts a wide variation in the topography of basal thermochemical structure margins, from near vertical to shallowly sloping. Subducted oceanic lithosphere reaches the lowermost mantle and then flows laterally towards the upwelling region. Basal thermochemical structures are characterized by high temperature and low viscosity (Supplementary Fig. S4). Colder and stiffer slabs push the thermochemical structures to form steep ridges along their margins, as observed in previous studies^2,6,46^. An illustrative example is the concave-upward tops of the superocean structure at 800 Ma (Fig. 6). It is notable that thermochemical ridges sometimes migrate toward the interior of the basal thermochemical structures, causing their steep edges to become smoother (Fig. 6 and Supplementary Fig. S5). This is likely because subducted slabs gradually become less negatively buoyant and less viscous (Supplementary Fig. S4) as they are heated by shear heating and by thermal diffusion. Over time, heated slabs may rise up from the CMB and climb over the edge of hot basal thermochemical structures, pushing outer, shallow ridges of thermochemical structures toward their interior, which smoothes the edges of hot basal thermochemical structures over time (Fig. 6 and Supplementary Fig. S5).

**Figure 7:**
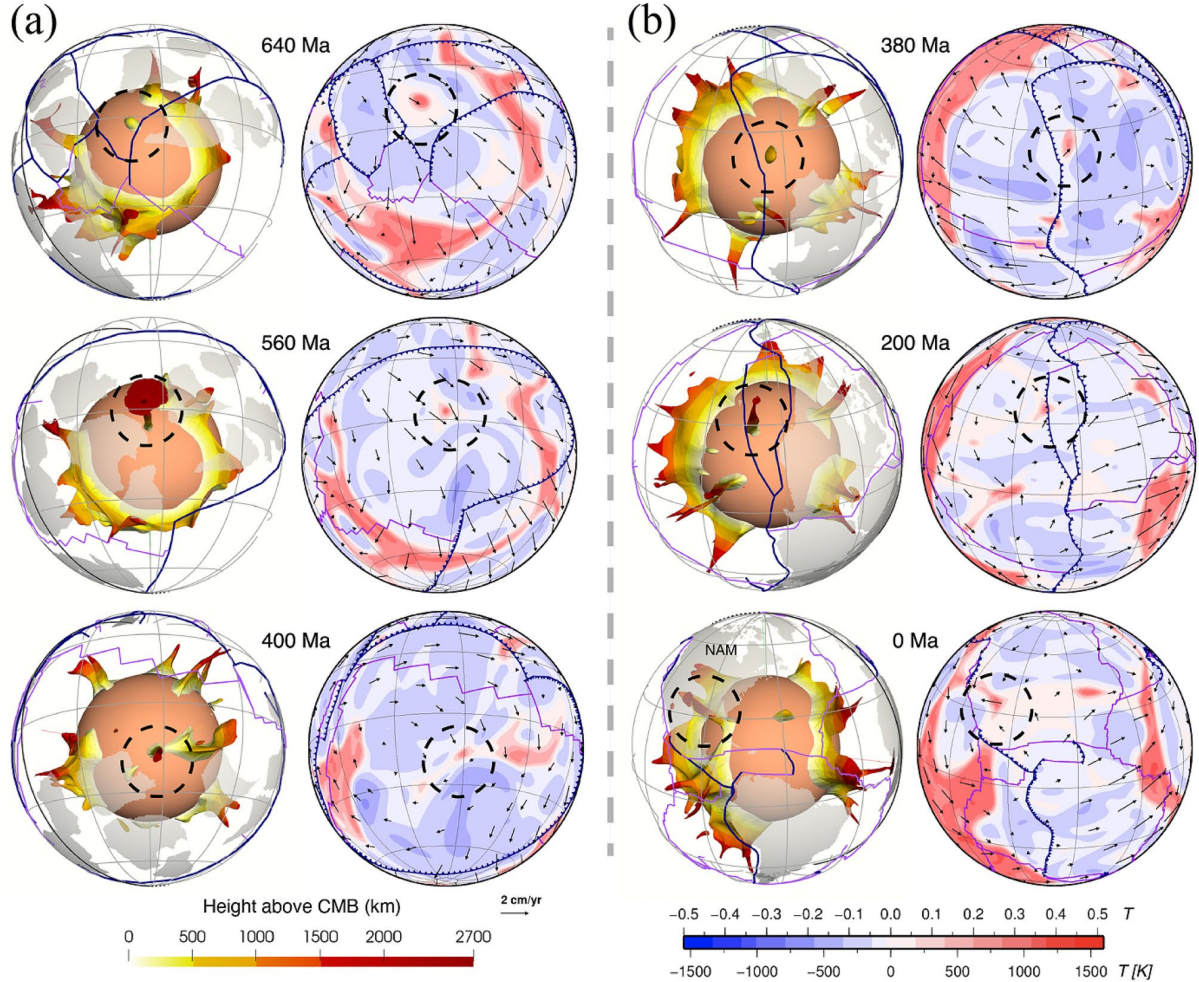
Formation of two distinct lone plumes (one in **(a)** and one in **(b)**, both marked by black dashed circles) due to **(a)** the sinking of a small network of slabs or **(b)** a small basal thermochemical structure is carved from a major thermochemical structure. In both **(a)** and **(b)**, the left panels show the thermochemical structures, the right panels show temperature anomaly at 2595 km depth. The lone plume in **(a)** merges with a small thermochemical structure at 400 Ma, and the lone plume in **(b)** connects to the northeastern Pacific thermochemical structure through two thin thermochemical ridges. The solid and dashed black meridians are longitudes 0º and 180º, respectively. *NAM* North America. Symbols are as in Fig. 2. This figure was created using the Generic Mapping Tools version 4.5.5 (https://www.generic-mapping-tools.org/)^85^ and ParaView version 5.8.1 (https://www.paraview.org/)^86^.

A link has long been proposed between hot spots and deep mantle plumes^47^, and numerical models have shown that instabilities in the lowermost thermal boundary layer lead to the formation of mantle plumes^48,49^. In our model, plumes mostly rise from thermochemical ridges, especially the ones with a height > 1000 km above the CMB. Plumes entrain some dense material from basal thermochemical structures over time (Fig. 3 and Supplementary Fig. S1), which could explain the primitive signature of ocean island basalts^4^. Previous thermochemical modelling studies showed that plumes could entrain dense material from basal thermochemical structures and/or from subducted oceanic crust^50,51^. We cannot verify the latter as oceanic crust is not tracked in our model. In contrast to the “plume generation zone” hypothesis^14^, which suggests that plumes only form from the stationary LLSVP margins, our model predicts that plumes rooted on thermochemical ridges migrate both with the basal thermochemical structures as a whole and sometimes toward the interior of mobile thermochemical structures. For example, the thermochemical ridge (and the plume rising from it marked by dashed circles in Fig. 6) from the southern edge of the superocean structure migrate northward due to slab push^52^ at a rate of ~ 1.13 cm/year between 800 and 640 Ma, which is relatively fast for the deep mantle. Similarly, the ridges (and the plume rising from it marked by dashed circles in Supplementary Fig. S5) migrate westward at a rate of ~ 0.75 cm/year between 380 and 200 Ma from the eastern edge of the superocean structure. The plumes are largely vertical due to relatively low viscosity and large velocities of plume material, but sometimes show some tilting due to fast plate motion and induced nearly horizontal mantle flow (Fig. 2), which is consistent with earlier studies^49,53^. We observe that a lone plume can develop outside the main thermochemical structures^54^ (Fig. 7). A lone plume could emerge inside of a small network of sinking slabs^9^ (Fig. 7a), or when a small basal thermochemical structure is carved from a major thermochemical structure by subducted slabs (Figs. 5 and 7b). In general, lone plumes eventually join a large thermochemical structure. The existence of lone plumes may explain why hotspot magmatism is not spatially limited to the LLSVPs^55^. The model predicts a lone plume below North America at present-day (Fig. 7b), not far from the location of the Yellowstone hotspot, suggesting that a lone plume could potentially account for Yellowstone volcanism^55^.

## Discussion

Our 4D images (3D space and time) illustrate how the thermochemical structure evolves in response to surface plate motion history. In agreement with previous studies^7,8^, our models suggests that subduction history plays a first-order role in shaping the deep mantle. Our model does not predict the alternation between degree-1 and degree-2 lower mantle structures proposed by Zhong et al.^36^, because continents are always present in our time-dependent model, whereas they were not present in the degree-1 statistically steady-state model of ref.^36^. Zhang et al.^8^ built mantle flow models kinematically driven by simplified plate motion models back to 450 Ma and found that, due to convergence between Laurussia and Gondwana, there was only one basal thermochemical structure in the Pacific hemisphere and beneath southern Gondwana during and shortly after the assembly of Pangea. Subsequent circum-Pangea subduction broke the large structure into two and pushed the structure below southern Gondwana to beneath Africa and thus formed degree-2 structures more than 100 Myr after the Pangea assembly. Our model confirms that Laurussia-Gondwana convergence could have broken the sub-supercontinent thermochemical structure into segmented structures. However, basal thermochemical structures are not pushed to the superocean hemisphere in our model, impeded by simultaneous convergence between the Proto-Pacific Ocean and Gondwana (a part of the subduction girdle). Aside from the considered time period (1 Gyr here and 450 Myr in ref.^8^), the main difference between our model and that of Zhang et al.^8^ is that, in our model, there are more slabs related to the convergence between the Proto-Pacific Ocean and Gondwana. This is likely caused by (1) a faster subduction rate of the Proto-Pacific Ocean beneath Gondwana in our plate reconstruction model, (2) a thicker subducting slab in our flow model in which slabs are progressively assimilated assuming a half-space cooling model (with maximum seafloor age 80 Myr, see “Methods”), as opposed to arising from plate velocities in Zhang et al.^8^.

In our plate reconstructions, supercontinents breakup and assemble dominantly through introversion, which is an end-member model. Li et al.^28^ proposed that successive supercontinent assemblies alternate between introversion (e.g., Nuna to Rodinia) and extroversion (Rodinia to Pangea), forming a longer (~ 1–1.5 Gyr) tectonic cycle that is twice the duration of a supercontinent cycle (∼500–700 Myr). Li et al.^28^ also hypothesized that a supercontinent dominantly assembled by introversion should inherit the pre-existing subduction girdle and degree-2 basal mantle structure, while a supercontinent dominantly assembled by extroversion should cause the destruction of the subduction girdle and the formation of a degree-1 lower mantle structure. Our results agree with the conceptual model of Li et al.^28^ in that the lower mantle structure is dominated by degree-2 structure at all times in an introversion scenario. Whether an alternation between degree-1 and degree-2 lower mantle convection planforms may occur in an extroversion scenario remains to be determined. We find the (super)continent thermochemical structure alternates between segmented and unified, following the breakup and assembly of supercontinents, with a time delay of the order of 200 Myr. In addition, material can be exchanged between thermochemical structures in different hemispheres in our model when a small basal structure is carved from one structure and merges with the other, which is consistent with earlier studies^2,10^.

Seismic studies have identified variations in the topography of the LLSVPs from gentle to steeply dipping^12,19^, and it has been proposed that the variation might be caused by (1) variations in LLSVP composition, or (2) the interaction between LLSVPs of single composition with slabs^12^. Frost and Rost^19^ found the northern and eastern edges of the Pacific LLSVP to be shallowly and steeply dipping, respectively, and they proposed that the steeper edge on the eastern side of the LLSVP could be due to its closer proximity to an active subduction zone, which results in slabs are colder and stiffer close to the CMB. Our model results show that this variation in hot basal thermochemical structure topography can be reproduced by the interaction of slabs with single-composition structures just above the CMB (Fig. 6 and Supplementary Fig. S5). This is consistent with the idea that colder slabs tend to drive the formation of the steep edges of thermochemical structures^19^. Importantly, we find that the slope of the edge of thermochemical structures is transient and dynamic, and that it decreases likely because the slabs that push it heat up as they interact with the edge of hot structures over time^6^ (Fig. 6).

Based on the concept of the plume generation zone, plumes are expected to predominantly rise from the edge of the LLSVPs. However, statistical analyses show that whether hotspots and reconstructed locations of LIPs and kimberlites are spatially correlated with LLSVPs in general or specifically with LLSVP margins cannot be distinguished^56,57^. Our model shows that plumes mostly migrate with thermochemical ridges, which is consistent with analogue experiments^58^. The plumes and thermochemical ridges initially develop along the edges of the basal thermochemical structures^48,53,59^ and sometimes migrate toward the interior of the structures. This is consistent with the occurrence of hotspots above the interior of the Pacific LLSVP^56^.

Here we only present one mantle flow model, while the morphologies of basal thermochemical structures are well-known to be sensitive to multiple model parameters^2,8,60–62^, such as the excess density of the denser basal layer, phase changes, and mantle viscosity structure^60,61^. The excess density (controlled by buoyancy ratio) plays a dominant role, and decreasing the density contrast between the thermochemical structure and surrounding mantle leads to more deformation^11,62,63^. Whether the LLSVPs are chemically distinct from the ambient mantle remains debated^2,3^. The LLSVPs have been proposed to be compositionally distinct based on an anticorrelation between shear wave and bulk sound velocity anomalies^23,64^, and the strong *Vs* gradients along the LLSVPs margins^14,20^. Studies of the splitting of Earth’s free oscillations^65^ suggest high-density anomalies of less than 1.7% and 1% at 2850 km and 2300 km, respectively, in LLSVP regions. Tidal tomography suggests that the positive density contrast might be as little as 0.5% (ref.^66^), and global tomography including Stoneley modes suggest that LLSVPs are purely thermal structures^67^. The buoyancy ratio we use here for thermochemical structures (B = 0.25) corresponds to an excess density equal to 1%, and results in predicted thermochemical structure with heights (~ 600–800 km) comparable to seismic observations. Previous laboratory or modelling studies have shown that the long-term (e.g., the age of the Earth) survival of the dense basal thermochemical structures require an excess density larger than ~ 2% (buoyancy ratio equal to 0.5, ref^68^), ~ 2.3% (refs.^59,69^) or 3% (ref.^70^) compared to the surrounding mantle. The persistence of the basal layer also depends on other parameters including the Rayleigh number and viscosity of the denser layer^59,60^. Our model shows some entrainment of dense material^59^ (Fig. 3 and Supplementary Fig. S1), implying that the denser layer would not persist over Earth’s entire history or could not be assumed as primordial. The excess density deduced from our models is not directly comparable to that deduced from seismic tomography. Indeed, the conversion of mantle temperature predicted by mantle flow models to seismic velocities and the application of a tomographic filter^3,71^ suggest that compositionally distinct basal thermochemical structures are not required to obtain sharp *Vs* gradients, and that the anticorrelation of bulk and shear velocities could be associated with the post-perovskite phase transition. However, the LLSVPs are generally proposed to reach a height of more than 700 km above the CMB^13,14,18^, which cannot be fully explained by the presence of a ~ 200–300 km thick post-perovskite layer^18,72^. Further seismic studies are needed to advance this debate. Imposing phase changes could also affect the evolution of basal thermochemical structures: a phase transition at 660 km depth would inhibit the mass exchange between the upper and lower mantles and contribute to the stability of basal thermochemical structures^60^, while a viscosity reduction owing to post-Perovskite phase transition in the lowermost mantle could promote subducted slabs to go deeper into the lower thermal boundary layer and further shape basal structures^50^. The mantle viscosity structure is also poorly constrained^73^, and the viscosity laws and structures used in mantle flow models also affect the predicted deep mantle evolution^46^. For example, increasing the temperature dependence of viscosity tends to decrease the viscous coupling between basal thermochemical structure and surround mantle: the larger the temperature dependence, the less viscously coupled the two materials are^50,74^. A larger compositional viscosity for basal thermochemical structures that makes them more viscous than ambient mantle would also contribute to their long-term survival and stability^6,46,62^.

Our models suggest that the configuration of tectonic plates is reflected in the configuration of basal thermochemical structures after up to one mantle transit time (the time it takes for slabs to sink from the surface to the CMB): sinking slabs are associated with downwellings that also shape basal thermochemical structures (Fig. 6). The transit time, which depends on the poorly known viscosity of the mantle, is uncertain. For instance, a larger lower mantle viscosity would cause longer slab transit times. Considering these uncertainties, our model was calibrated to match available constraints^11^: the selected Rayleigh number and viscosity structure result in a transit time (~ 160–240 Myr^38^) consistent with independent constraints from tectonic reconstructions and seismic tomography^43,44^, and the chosen buoyancy of the basal layer results in predicted basal thermochemical structures (e.g., shape, height and edge topography) consistent with LLVSPs imaged by seismic tomography. The model results show that the topography of basal thermochemical structures and distribution of mantle plumes are transient^10^ and evolve dynamically with surface plate motions^2,7–9,75^.

## Methods

### Plate reconstructions

In a previous study^38^, we built three synthetic 1 Gyr plate reconstructions with the same relative plate motion but with different absolute reference frames. Here we choose the preferred reconstruction with a no-net-rotation frame (Fig. 1, reconstruction NNR in ref.^38^), in which the net lithospheric rotation is removed. The relative plate motion model includes a synthetic plate motion model between 1000 and 250 Ma that we link to a published palaeogeographical plate reconstruction^40^ for 250–0 Ma. For the synthetic part of the plate motion model (1000–250 Ma), we only use 15 major cratons before 410 Ma, and we add the Amuria plate at 410 Ma.

We consider three supercontinents that breakup and reassemble by predominantly introversion. The first supercontinent assembles at 900 Ma and breaks up at 800 Ma (to mimic Rodinia^28^). Then, all tectonic blocks reassemble to form the second supercontinent at 620 Ma (to mimic Pannotia^30^), mostly through the closure of young interior oceans that opened during the breakup of the first supercontinent. The second supercontinent breaks up at 600 Ma. The last supercontinent-Pangea assembles at 320 Ma and lasts until 200 Ma. A circum-(super)continent subduction girdle exists at all times, largely separating Earth into a superocean hemisphere and a (super)continent hemisphere.

All major tectonic plates move relative to either Laurentia or Africa via plate motion chains (except oceanic plates in the superocean that move relative to a triple junction within that ocean), which ultimately move relative to the spin axis via a no-net-rotation reference frame. Globally averaged root-mean-square plate velocities for the plate motion model fluctuate between 4 and 6 cm/year most of the time, which is comparable to plate velocities over the last 200 Myr (ref.^76^). Trench orthogonal migration rates are generally below 10 cm/year, with slightly more slab retreat than advance, which is geodynamically reasonable^77^. The trench orthogonal convergence rate before 250 Ma is generally ~ 4–5 cm/year, which is comparable to the present-day rate^38^.

### Mantle flow model

Here we select one mantle flow model constrained by the plate reconstruction NNR from our previous study (case NNR_WU in ref.^38^). The model uses the extended-Boussinesq approximation in a version of CitcomS^78^ that has been modified for progressive assimilation of surface boundary conditions from plate reconstructions^79^. We build the thermal structure of the lithosphere using reconstructed seafloor ages and a half-space cooling model to a depth of 2.32 $\sqrt{A{\kappa }_{0}}$, where A is the seafloor age, and *κ*_*0*_ = 1e−6 m^2^ s^−1^ is the thermal diffusivity. The maximum seafloor age is set to 80 Myr (for which the lithosphere is ~ 120 km thick) to mimic the plate cooling model. Similarly, we build the thermal structure of subducting slabs using seafloor ages at 1 Myr intervals specifying a 45° dip angle to a maximum depth of 350 km. The thermal structures of the lithosphere and subducting slabs are built at 1 Myr intervals, and blended with the solution of the convection equations at each numerical time step through linear interpolation. A detailed description of the method can be found in ref.^79^. We apply an isothermal (T = 273 K) and kinematic^80^ (plate velocities exported from the reconstruction NNR at 1 Myr intervals) boundary condition at the surface, and an isothermal (T = 3,373 K) and free-slip boundary condition at the CMB. The model consists of ~ 13 million nodes (129 × 129 × 65 × 12), with radial mesh refinement to obtain slightly higher resolutions at the surface (∼50 × 50 × 15 km) and CMB (∼28 × 28 × 27 km), and a lower resolution in the mid-mantle (∼40 × 40 × 100 km).

Slabs are initially built from surface to 550 km depth (with dip angles of 45° above 425 km depth and 90° below 425 km depth, Supplementary Fig. S6). The 1000 Ma plate configuration was applied during a 200 Myr warm-up phase before the main calculation, during which ocean floor is continuously subducted along fixed subduction zones. The initial condition includes a 113-km-thick denser basal layer (2% of the volume of the mantle) of excess density 1%. The excess density is defined by the buoyancy ratio B = δρ_ch_/(ραΔT) = 0.25, where ρ = 5546 kg m^−3^ is the density (the average value of the bottom 100 km above the CMB from Preliminary Reference Earth Model^81^), α = 1.32 K$\times {10}^{-5}$ ^−1^ is the coefficient of thermal expansivity (the average value of the bottom 100 km above the CMB, Supplementary Fig. S7), ΔT = 3,100 K is the temperature difference between surface and CMB, δρ_ch_ = 56.8 kg m^−3^ (a result of the values listed above) is density contrast disregarding thermal effects. The composition field is tracked with tracers using the ratio tracer method^63,82^.

The convective vigour is controlled by the Rayleigh number:$Ra={\alpha }_{0}{\rho }_{0}{g}_{0}\Delta T{h}_{M}^{3}/{\kappa }_{0}{\eta }_{0}=7.8\times {10}^{7}$, where *α*_*0*_ = 3 K$\times {10}^{-5}$^−1^ is the reference coefficient of thermal expansivity at the surface, *ρ*_*0*_ = 4000 kg m^−3^ is the reference density, *g*_*0*_ = 9.81 m s^−2^ is the acceleration of gravity on Earth's surface, and *h*_*M*_ = 2867 km is the thickness of the mantle. The dissipation number: $Di={\alpha }_{0}{g}_{0}{R}_{0}/{C}_{{P}_{0}}=1.56$, where ${C}_{{P}_{0}}$= 1200 J kg^−1^ K^−1^ is the reference heat capacity. The viscous dissipation for depths less than 300 km is ignored because surface plate motions are imposed^48^. As in Flament^11^, the rate of internal heating for the whole model is *H* = 33.6 TW. The coefficient of thermal expansion depends on depth and temperature (Eq. 2 of^83^): *α(T, z)* = *(b*_*0*_ + *b*_*1*_*T* + *b*_*2*_*T *^*−2*^*) exp(− b*_*3*_*z)*, where *T* is the temperature, *z* is the depth, and *b*_*0*_ = 2.68 $\times {10}^{-5}$, *b*_*1*_ = 2.77 $\times {10}^{-9}$, *b*_*2*_ = −1.21 and *b*_*3*_ = 3.76 $\times {10}^{-7}$ are coefficients obtained from the inversion of thermodynamic data^83^ (Supplementary Fig. S7).

Viscosity is temperature, composition and depth dependent:

$$\eta =\eta \left(r\right){ \eta }_{0}{ \eta }_{\mathrm{C}}\mathrm{exp}\left(\frac{{E}_{\eta }+{\rho }_{0}g{Z}_{\eta }({R}_{0}-r)}{R(T+{T}_{off})}-\frac{{E}_{\eta }{+{\rho }_{0}gZ}_{\eta }({R}_{0}-{R}_{c})}{{R(T}_{CMB}+{T}_{off})}\right)$$

where η(r) is a depth-dependent pre-factor with values 0.02, 0.002, 0.02, 0.2 defined with respect to the reference viscosity (*η*_*0*_ = 1.1e21 Pa s) for mantle above 160 km, between 160 and 310 km, between 310 and 660 km and below 660 km, respectively. *η*_*C*_ is the compositional viscosity pre-factor: 1, 100, 10 for ambient mantle, continental lithosphere and basal layer, respectively, in the initial condition. *E*_*η*_ = 283.5 kJ mol^−1^ is the activation energy, *Z*_*η*_ = 2.1 cm^3^ mol^−1^ is the activation volume, *g* is the acceleration of gravity, *R*_*0*_ = 6371 km is the radius of the Earth, *r* is the radius, *R* = 8.31 J mol^−1^ K^−1^ is the universal gas constant, *T* is the temperature, *T*_*off*_ = 496 K is a temperature offset, and *R*_*C*_ = 3504 km is the radius of the core. *E*_*η*_ and *T*_*off*_ are selected to obtain viscosity variations by three orders of magnitude as a function of temperature^11^ (Supplementary Fig. S7). We limit the viscosity field to values between 1.1 Pa$\times {10}^{20}$ s and 2.2 Pa$\times {10}^{23}$ s (i.e., viscosity varies by up to a factor of 2000), because large viscosity variations are challenging to resolve numerically^11,48^. In addition, the Rayleigh number and viscosity-related parameters are chosen to obtain slab sinking rates that are comparable to that inferred from global tectonic reconstructions and seismic tomography^11^. The CMB temperature (3373 K) in our model is slightly higher than the lower bound of 3300–4300 K estimated by Lay et al.^84^.

In post-processing, we regard mantle hotter than layer average by non-dimensional value 0.1 (310 K) as hot basal thermochemical structure (except in Fig. 3, in which the thermochemical structure is defined as mantle consisting of at least 50% dense material), and mantle colder than layer average by non-dimensional value 0.05 (155 K) as slabs.

## Supplementary Information

Supplementary Figures.

Supplementary Video 1.

Supplementary Video 2.

## Data Availability

The mantle flow model results that are readable in ParaView are available for download at https://doi.org/10.5281/zenodo.4710580. The synthetic plate tectonic model (reconstruction NNR) is available for download at https://doi.org/10.5281/zenodo.3854459. The version of *CitcomS* used in this research is available from https://github.com/EarthByte/citcoms (commit hash: 6f0f654793705af828b3916ba87c91463fc17673).
