# Supplementary material for: The evolution of basal mantle structure in response to supercontinent aggregation and dispersal
Source: Sci Rep. 2021 Nov 25;11:22967. doi: 10.1038/s41598-021-02359-z (PMC8617165; doi:10.1038/s41598-021-02359-z)
Supplement: Supplementary file 1 — Supplementary Figures. [file 41598_2021_2359_MOESM1_ESM.docx]

Supporting Information for

**The evolution of basal mantle structure in response to supercontinent aggregation and dispersal**

Xianzhi Cao^1,2,3^, Nicolas Flament^3^, Ömer F. Bodur^3^, R. Dietmar Müller^2^

^1^Frontiers Science Center for Deep Ocean Multispheres and Earth System; Key Lab of Submarine Geosciences and Prospecting Techniques, MOE and College of Marine Geosciences, Ocean University of China, Qingdao 266100, China

^2^EarthByte Group, School of Geosciences, The University of Sydney, Sydney, New South Wales, Australia

^3^GeoQuEST Research Centre, School of Earth and Environmental Sciences, University of Wollongong, Northfields Avenue, NSW 2522 Australia

# Supplementary figures and captions


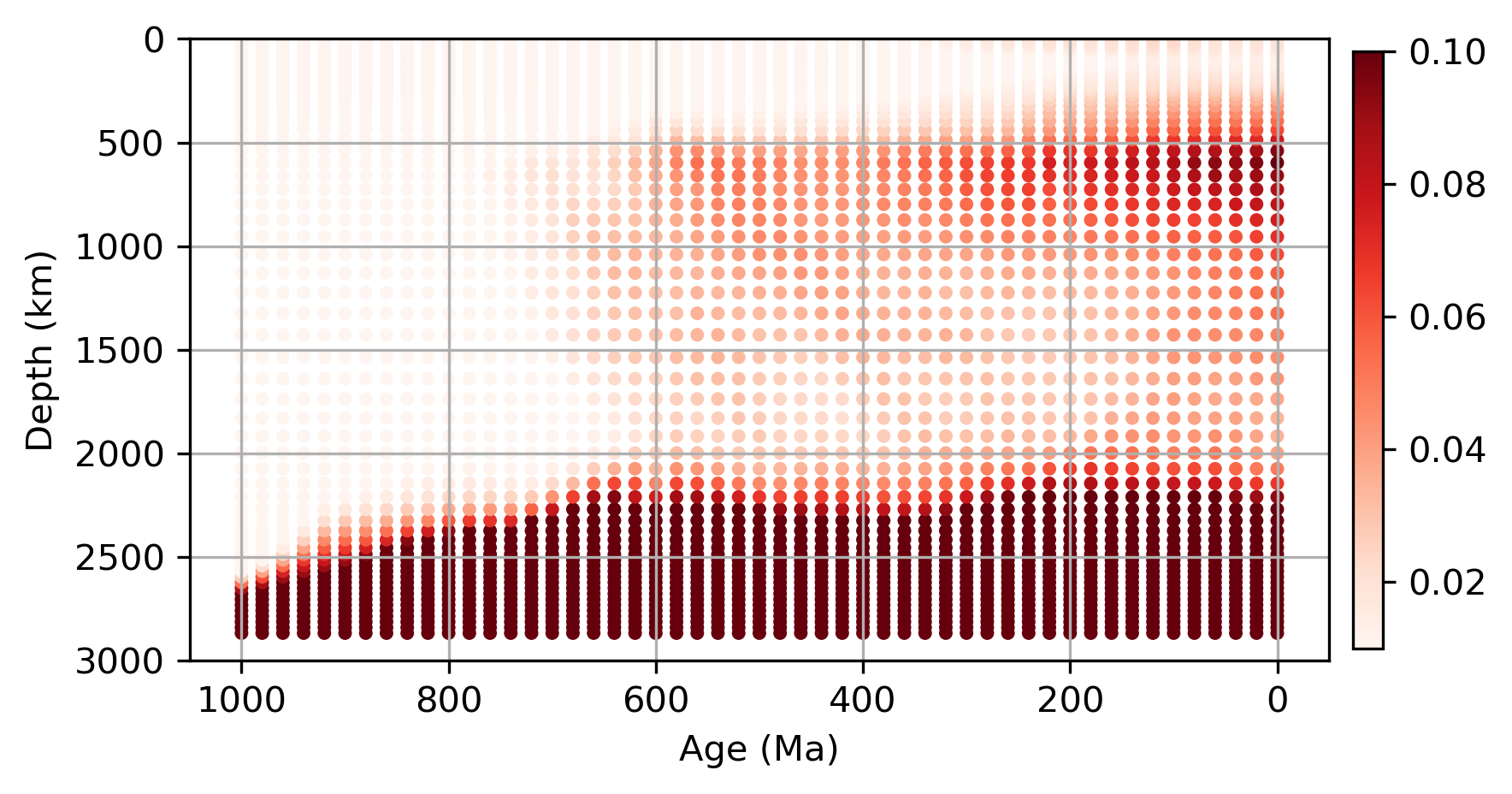


Figure S1. Average relative concentration of dense material (originally just above the core-mantle boundary) as a function of depth and time. For a given age, concentrations are normalised by the largest value across all depths so that the largest concentration at each time step is equal to one. This figure was created using matplotlib version 3.2.2 (https://matplotlib.org/)^1^.


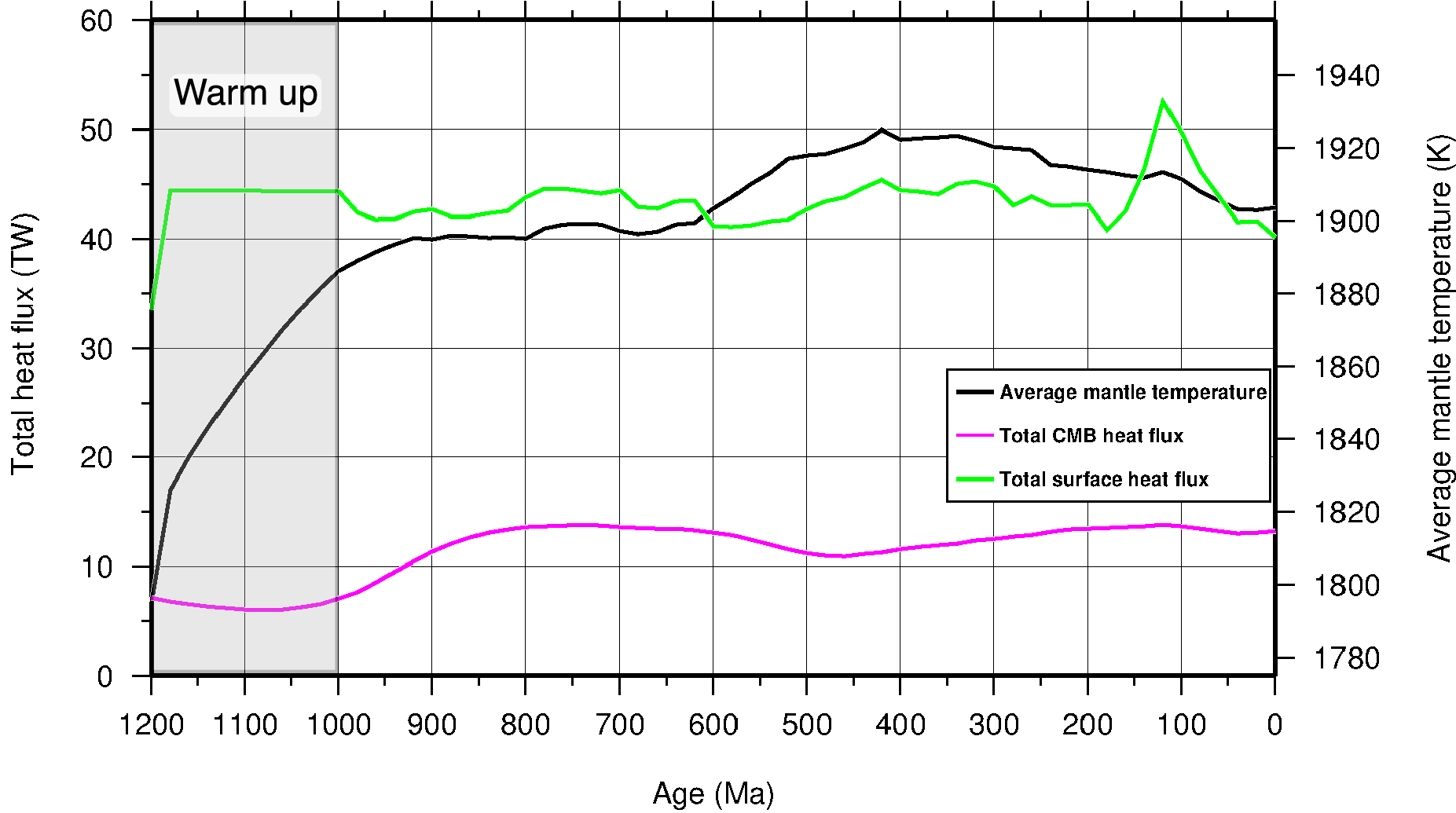


Figure S2. Time-dependent global heat flux at the core-mantle boundary and surface, and average mantle temperature through time. Gray shading denotes the model warm up phase. CMB: core-mantle boundary. This figure was created using the Generic Mapping Tools version 4.5.5 (https://www.generic-mapping-tools.org/)^2^.


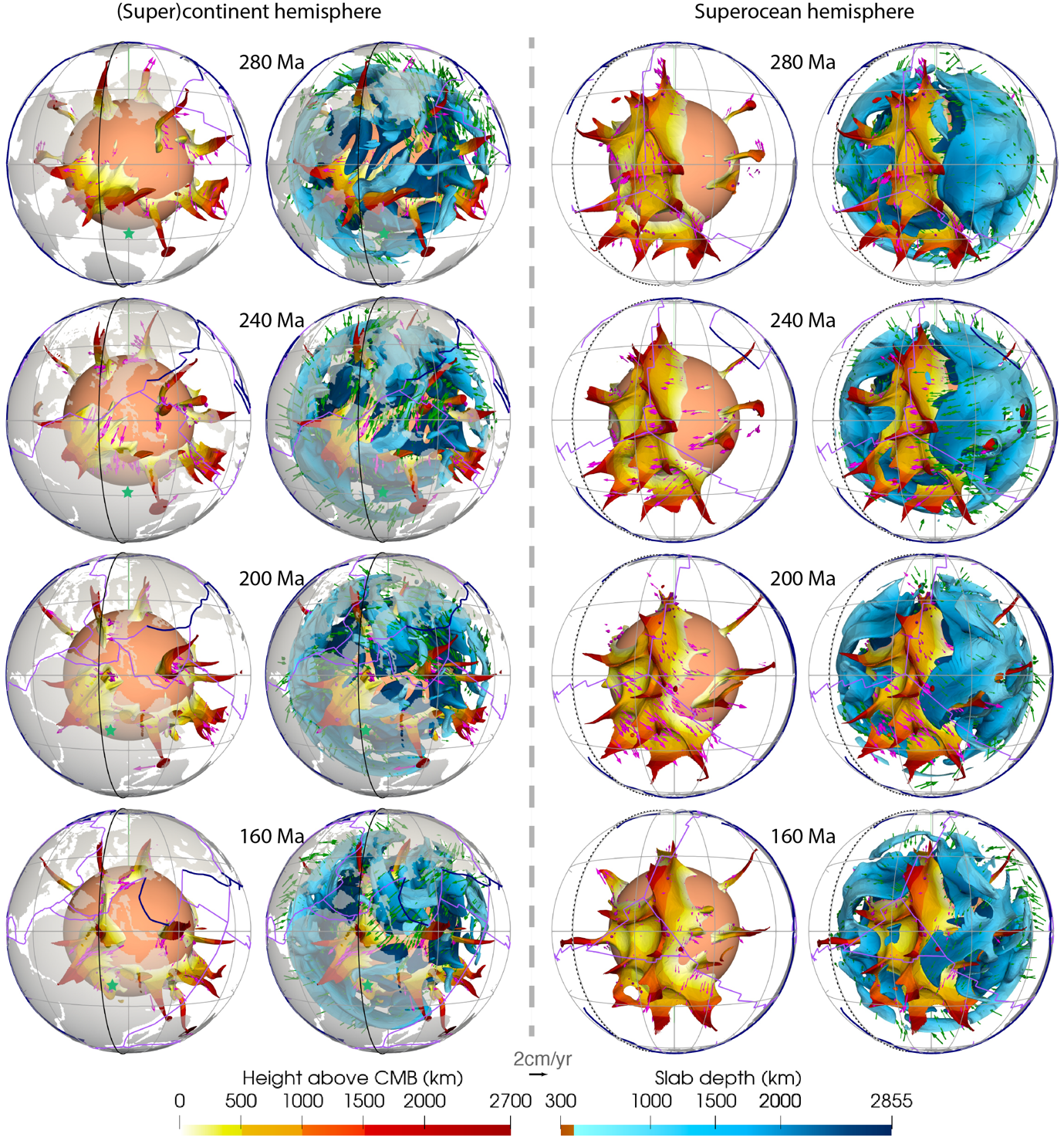


Figure S3. Evolution of the basal mantle structures in two hemispheres since 280 Ma in 40 Myr increments. Basal structures are coloured by height above CMB (left colour scale), and subducted slabs are coloured by depth (right colour scale). Slabs deeper than 1000 km are shown. Dark blue lines denote subduction zones, purple lines denote ridges and transforms. Magenta and green arrows show the velocity of the basal structures and slabs, respectively. The grey lines are graticules. The solid and dashed black meridians denote longitudes 0º and 180º, respectively. This figure was created using ParaView version 5.8.1 (https://www.paraview.org/)^3^.


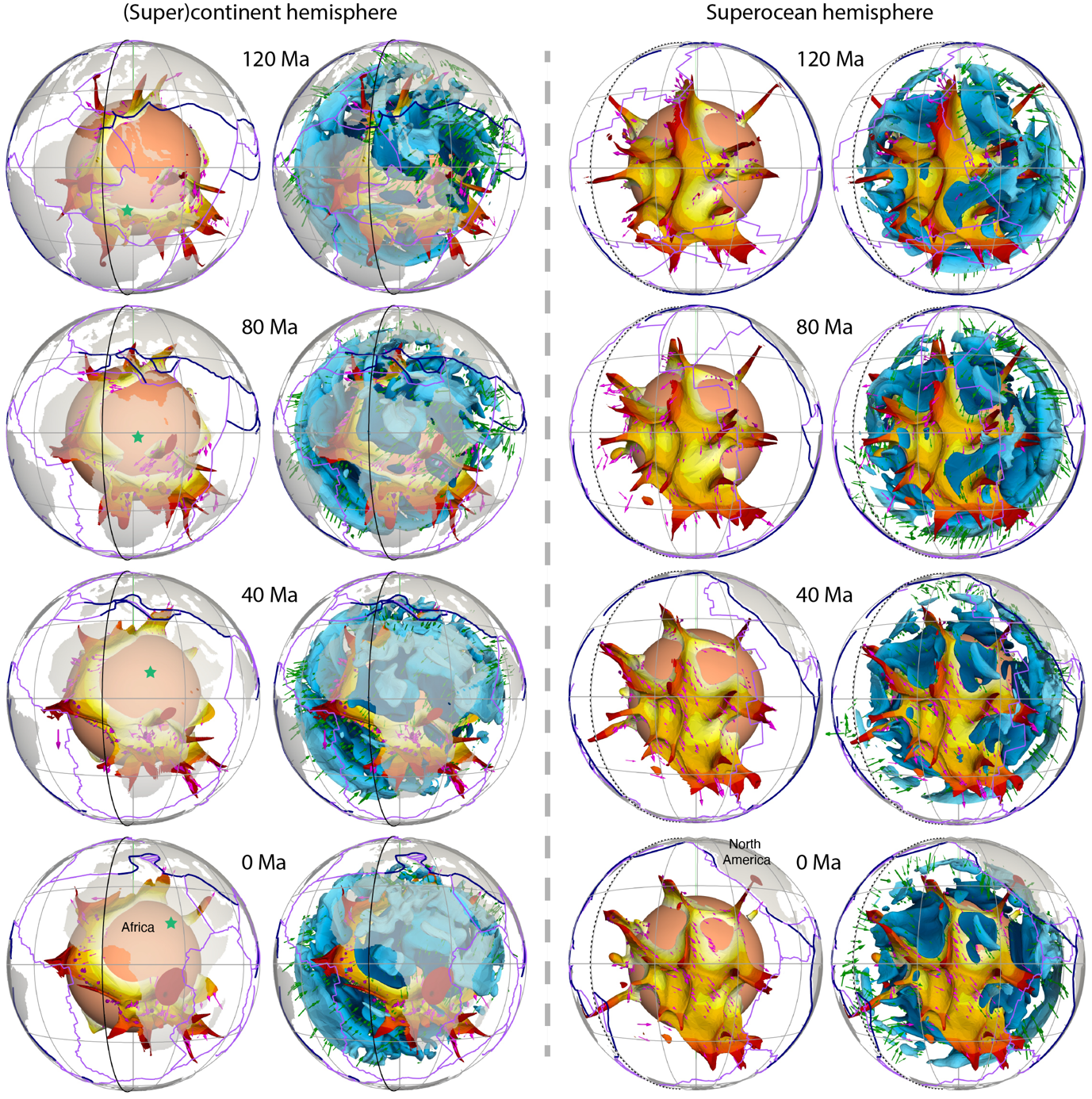


Figure S3. *Continued*.


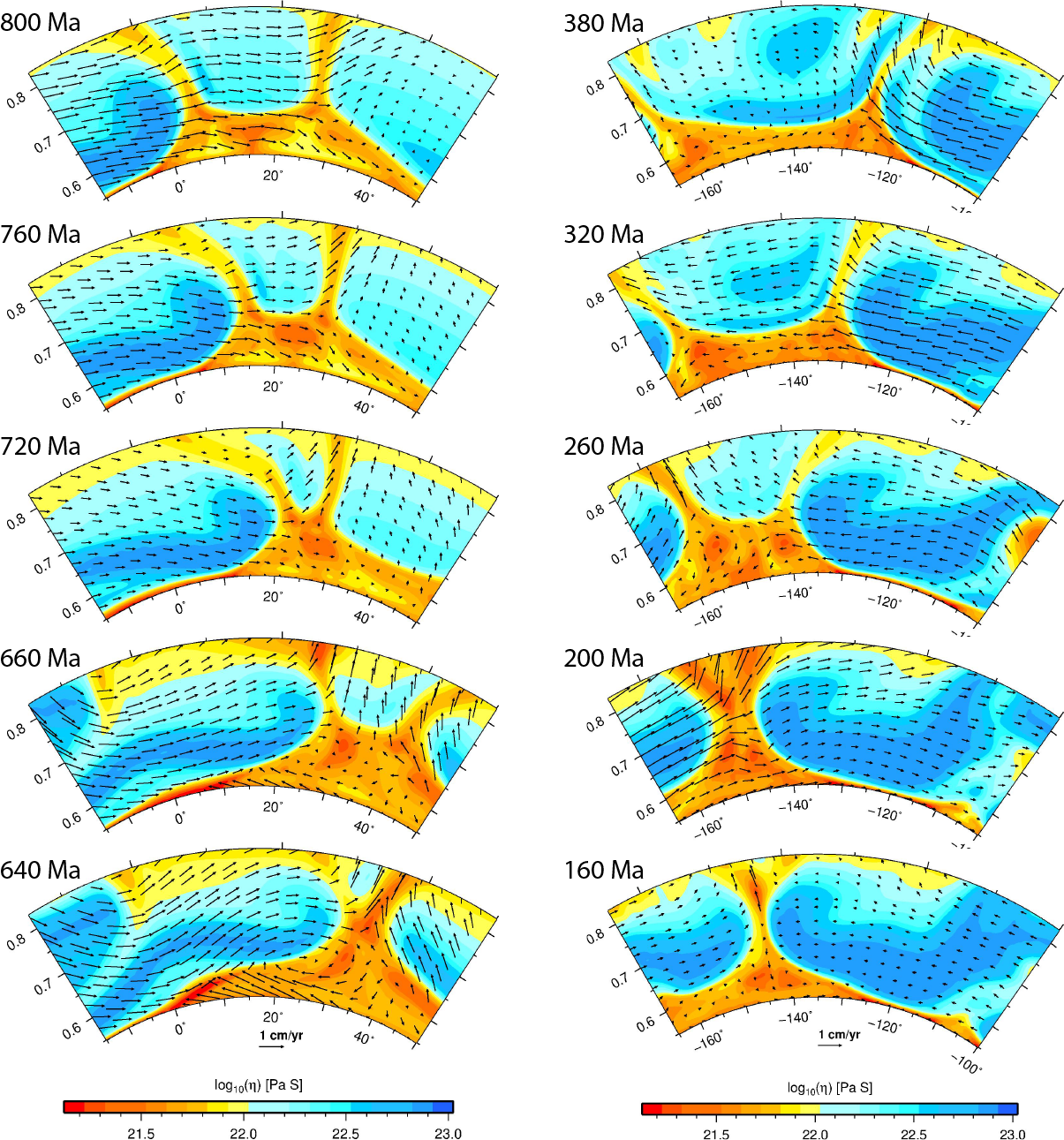


Figure S4. The left panel shows viscosity field along a North-South-trending cross section shown in Figure 6, the right panel shows viscosity field along an east-west-trending cross section shown in Figure S5. This figure was created using the Generic Mapping Tools version 4.5.5 (https://www.generic-mapping-tools.org/)^2^.


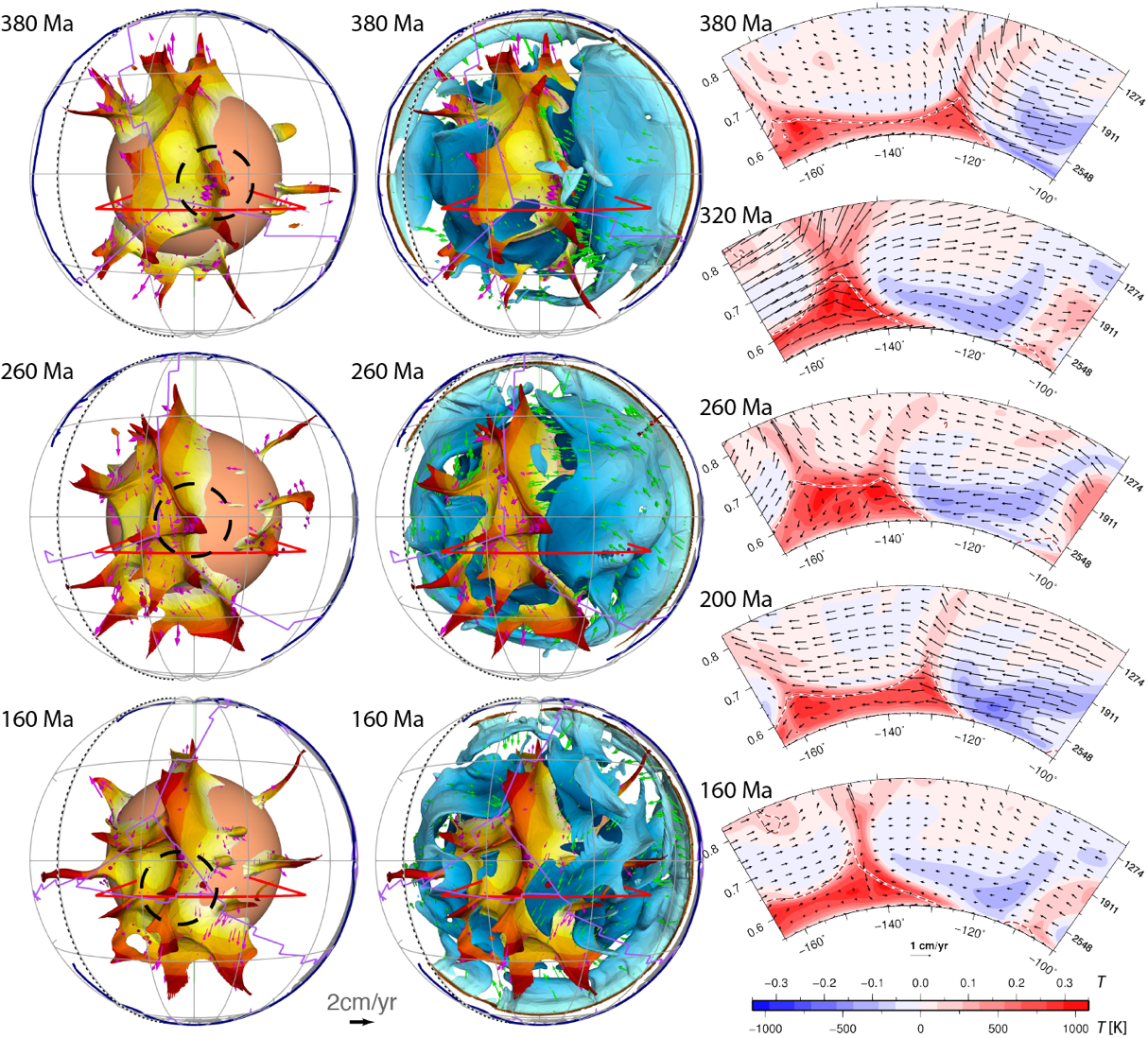


Figure S5. Edge migration of the superocean basal structure between 380-160 Ma. The left panel shows the basal structures, the middle panel shows both the basal structures and slabs (blue), the right panel show the temperature anomaly (temperature field with layer average removed) along an east-west-trending cross-section. The locations of the cross-section are shown as red lines in the left and middle panels. Green and magenta arrows in the left and middle panels show the velocity of the slabs and basal structures, respectively. The brown dashed contours in the right panel indicate 50% concentration of dense material. This figure was created using the Generic Mapping Tools version 4.5.5 (https://www.generic-mapping-tools.org/)^2^ and ParaView version 5.8.1 (https://www.paraview.org/)^3^.


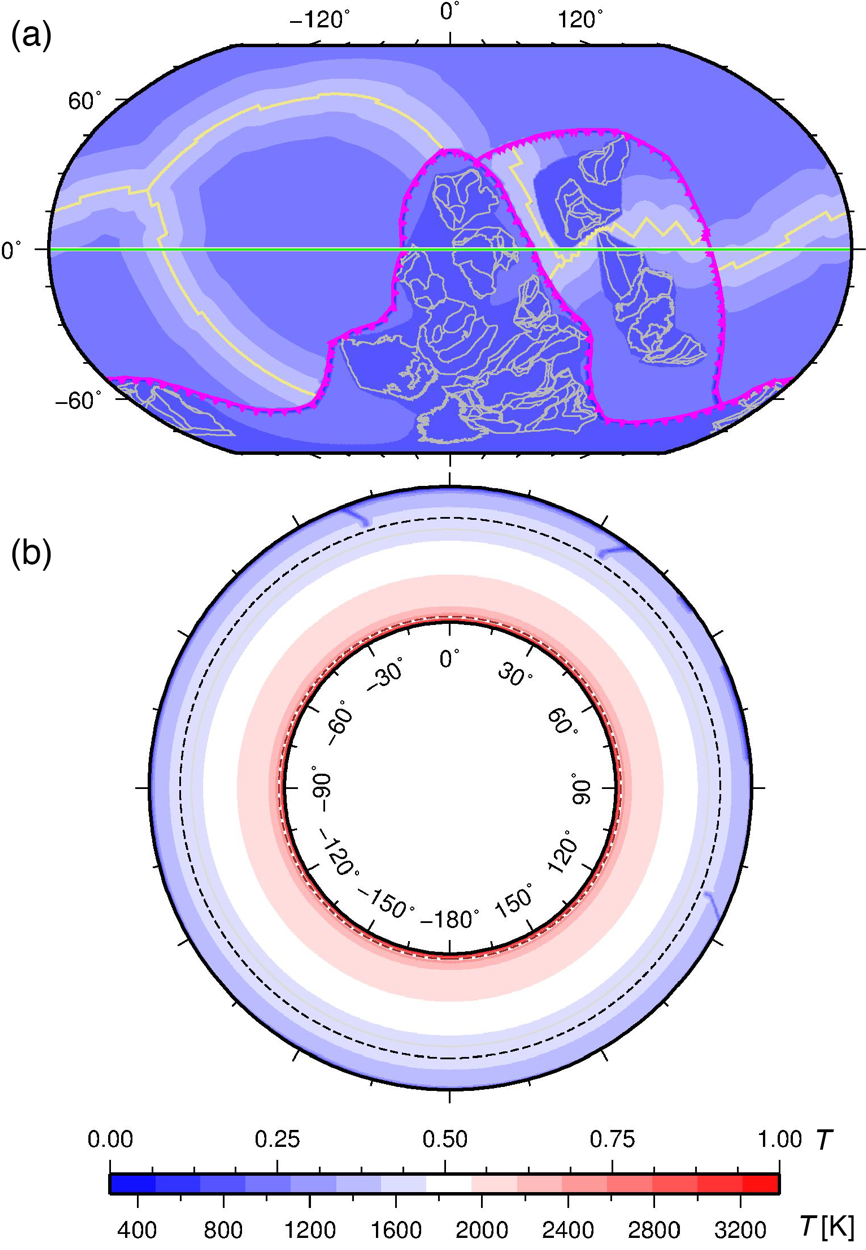


Figure S6. Initial mantle temperature for the mantle flow model at 1200 Ma. (a) Temperature at 60 km depth. Mid-ocean ridges and transform faults are shown as yellow lines, subduction zones as magenta lines with triangles on the overriding plate, reconstructed coastlines and sutures as grey polygons. (b) Whole mantle cross-section along the equator (green line in a). The dashed black line is the upper-lower-mantle boundary, and the dashed brown line outlines the compositionally distinct basal layer. The numbers above the colour scale denote non-dimensional temperature, and the numbers below the colour scale denote dimensional temperature. This figure was created using the Generic Mapping Tools version 4.5.5 (https://www.generic-mapping-tools.org/)^2^.


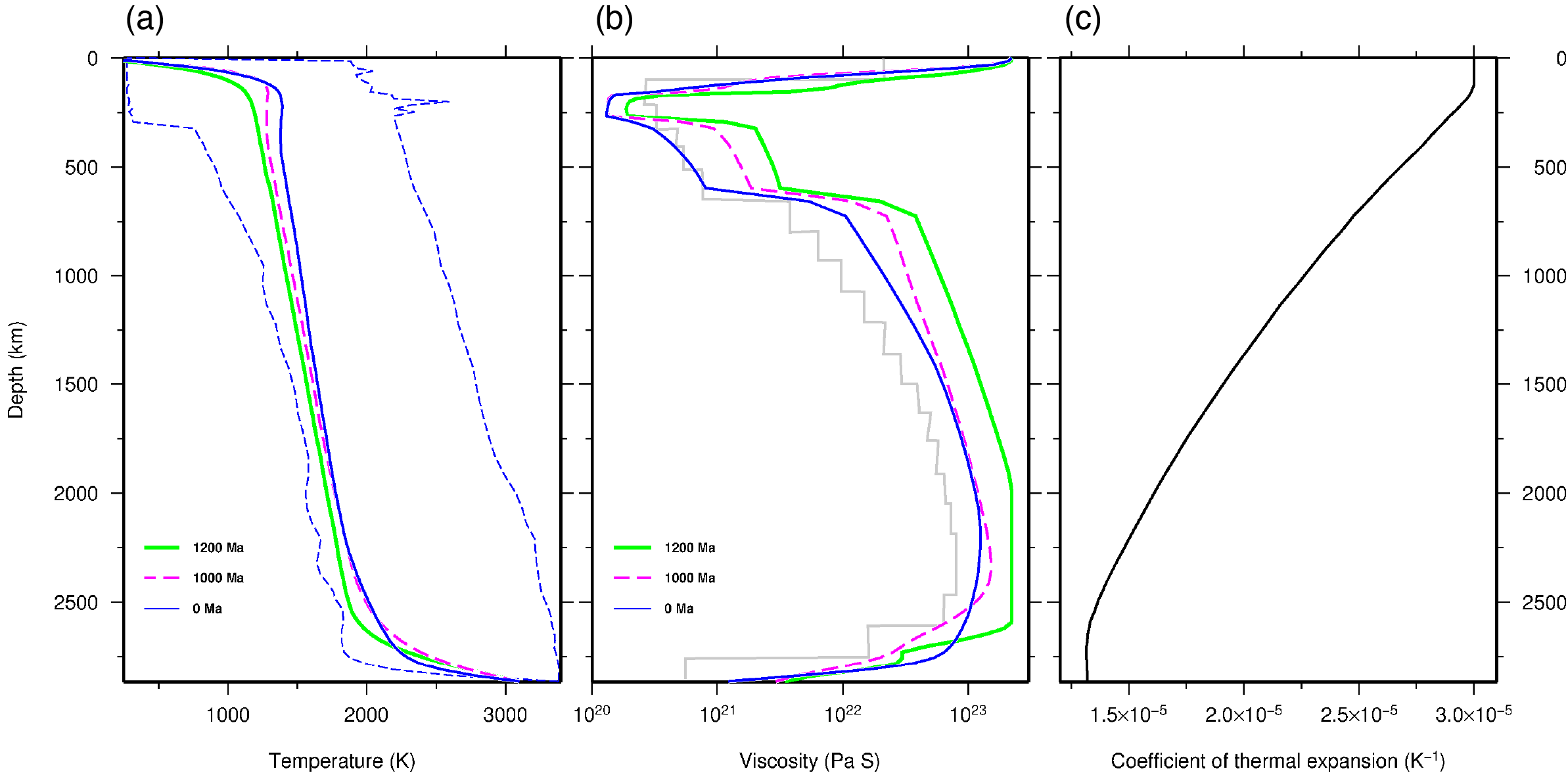


Figure S7. Model parameters or calculated results for the mantle flow model. (a) Horizontally-averaged temperature at 1200 Ma, 1000 Ma and 0 Ma. The blue dashed lines show the minimum and maximum temperatures at the present-day. (b) Horizontally-averaged viscosity at 1200 Ma, 1000 Ma and 0 Ma. The grey line shows the viscosity profile estimated by Steinberger & Calderwood (2006). (c) Depth-dependent coefficient of thermal expansion. This figure was created using the Generic Mapping Tools version 4.5.5 (https://www.generic-mapping-tools.org/)^2^.

# Captions for the supplementary animations

Supplementary Animation 1. Evolution of mantle temperature in the (super)continent hemisphere since 1000 Ma. The left panel shows basal structures (310 K hotter than ambient mantle), the right panel shows both basal structures and slabs (155 K colder than ambient mantle). Dark blue lines denote subduction zones, purple lines denote ridges and transforms, and magenta and green arrows show the velocity of the basal structures and slabs, respectively. The solid black meridian denotes longitudes 0º. This animation was created using Adobe Premiere Pro CC 2017.

Supplementary Animation 2. Same as Supplementary Animation 1, except for the superocean hemisphere. The dashed black meridian denotes longitudes 180º. This animation was created using Adobe Premiere Pro CC 2017.

# References

1 Hunter, J. D. Matplotlib: A 2D graphics environment. *Computing in science & engineering* **9**, 90-95 (2007).

2 Wessel, P., Smith, W. H., Scharroo, R., Luis, J. & Wobbe, F. Generic mapping tools: improved version released. *Eos, Transactions American Geophysical Union* **94**, 409-410 (2013).

3 Ayachit, U. *The paraview guide: a parallel visualization application*. (Kitware, Inc., 2015).
